# Extensive cytokine biomarker analysis in serum of Guillain-Barré syndrome patients

**DOI:** 10.1038/s41598-023-35610-w

**Published:** 2023-05-23

**Authors:** Xiaocong Li, Liping Yang, Guowei Wang, Yanping Yuan, Na Wei, Wanqiu Yang, Xiaoli Wang, Zhenhai Wang

**Affiliations:** 1 Ningxia Medical University, Yinchuan, 750004 Ningxia China; 2 Institute of Medical Sciences, General Hospital of Ningxia Medical University, Yinchuan, 750004 Ningxia China; 3 Diagnosis and Treatment Engineering Technology Research Center of Nervous System Diseases of Ningxia Hui Autonomous Region, Yinchuan, 750004 Ningxia China; 4 The No.1 People’s Hospital of Shizuishan, Shizuishan, 753200 Ningxia China

**Keywords:** Lab-on-a-chip, Cytokines, Neurological disorders

## Abstract

Guillain-Barré syndrome (GBS) is an acute idiopathic polyneuropathy which is related to infection and immune mechanism. The exact pathogenesis of the disease is unknown and treatment is limited. Thus, the purpose of the study is to identify biomarkers of GBS serum and elucidate their involvement in the underlying pathogenesis of GBS that could help to treat GBS more accurately. Antibody array technology was used to detect the expression levels of 440 proteins in serum of 5 GBS group and 5 healthy control group. Sixty-seven differentially expressed proteins (DEPs) were identified by antibody array, among which FoLR1, Legumain, ErbB4, IL-1α, MIP-1α and IGF-2 were down-regulated, while 61 proteins were up-regulated. Bioinformatics analysis indicated that most DEPs were associated with leukocytes, among which IL-1α, SDF-1b, B7-1, CD40, CTLA4, IL-9, MIP-1α and CD40L were in the center of protein–protein interaction (PPI) network. Subsequently, the ability of these DEPs to distinguish GBS from healthy control was further evaluated. CD23 was identified by means of Random Forests Analysis (RFA) and verified by enzyme-linked immunosorbent assay (ELISA). The ROC curve result of CD23 respectively displayed that its sensitivity, specificity and AUC were 0.818, 0.800 and 0.824. We speculate that activation of leukocyte proliferation and migration in circulating blood might be associated with inflammatory recruitment of peripheral nerves, leading to the occurrence and development of GBS, but this conclusion still requires deeper confirmation. More importantly, central proteins may play a pivotal role in the pathogenesis of GBS. In addition, we detected IL-1α, IL-9, and CD23 in the serum of GBS patients for the first time, which may be promising biomarkers for the treatment of GBS.

Guillain-Barré syndrome (GBS) is an immune-mediated acute inflammatory peripheral neuropathy, which is characterized by symmetric delayed paralysis of limbs and the disappearance or weakening of tendon reflexia^1^. The prevalence of the disease is reported to be 1–2 cases per 100 000 people per year^2^. Currently, the treatment of GBS is intravenous immunoglobulin or plasma exchange, and there is no specific treatment for this disease. Studies have reported that most patients have a relatively good prognosis after immunotherapy, but serious disability remains in about 20% of cases, and about 5–10% of patients die^3,4^.

The exact etiology of GBS is unknown. Studies have shown that 2/3 of patients have symptoms of respiratory or gastrointestinal infection before onset^5^. A number of infectious agents have been identified, including Campylobacter jejuni, cytomegalovirus, Epstein-Barr virus, and hepatitis C and E viruses^6^. Recently, increasing evidence has shown that GBS may also be associated with Zika^7^, chikungunya, dengue, and Japanese encephalitis virus infections^8^. There is scientific evidence to support an autoantibody-mediated immune process, triggered by molecular mimicry between gangliosides on the cell membrane of peripheral nerves and the microorganism^9^, including recent insect-borne infections^10^. Although some studies have proposed that immune responses such as activation of macrophages, complement systems, and T cell-mediated cytotoxicity lead to demyelination and axonal damage in the peripheral nervous system, further leading to the development of GBS, the exact pathogenesis remains unclear^11–13^. As is known to all, the combination of a variety of cytokines in the differentiation and activation of immune cells like B lymphocytes, T lymphocytes, and macrophages, plays an important role. Therefore, cytokines are important upstream and downstream mediators in many inflammatory diseases. In fact, many studies have reported the involvement of a complicated cytokine system in the pathogenesis of GBS by promoting or suppressing inflammation or by double action. Pro-inflammatory cytokines that have been proved to take a key role in the induction of GBS are IFN-γ, TNF-α and interleukin (IL)-6, -17, -22 and -23^14–17^. TNF-α and IFN-γ levels increased during the acute phase of GBS,but decreased during the recovery period, suggesting that they may be related to the progression of GBS^18,19^. Anti-inflammatory cytokines such as IL-4, IL-10 and TGF-β may have a role in reducing inflammation response^14,15^, whereas IL-27 has an initial pro-inflammatory effect and then an anti-inflammatory effect during recovery in GBS^17^. Besides, cytokines can also be used as biomarkers for differential diagnosis. Gautier Breville et al. reported that the expression of IL-8 in cerebrospinal fluid (CSF) helped to distinguish acute inflammatory demyelinating polyneuropathy type (AIDP-type) GBS from chronic inflammatory demyelinating polyneuropathy (CIDP) with high specificity and positive predictive value^20^. Although it has been determined that these biomarkers are related to GBS, only a few of them have been used in clinical application. Therefore, searching for new biomarkers is a way to develop new drugs for GBS.

In the current study, the cytokines of GBS serum were detected by high-throughput antibody array technology, and the enrichment analysis of GO term and KEGG were used to further elucidate the potential pathogenesis involved in GBS, which provided the basis for finding the exact therapeutic target.

## Results

### Clinical characteristics of the study population

At the screening stage, there were 5 cases in the GBS group, with an average age of 50.80 ± 18.42 (3 males and 2 females, ranging in age from 20 to 69 years). Similarly, 5 healthy controls (3 males and 2 females, aged from 23 to 63 years, mean age 49.20 ± 15.56 years) were enrolled. At the verification stage,there were 15 GBS patients with an average age of 56.70 ± 11.17 years (8 males and 7 females, aged 29–70 years). The average age of the healthy control (HC) group was 55.18 ± 14.84 (7 males and 4 females, aged from 23 to 75 years). In this cohort, 5 subjects from the screening phase were enrolled (4 in the GBS group, including 2 males and 2 females, and 1 female in the healthy control group). Table 1 presents the clinical features of patients with GBS.

**Table 1:** Demographic, clinical characteristics, and laboratory results of GBS.

Demographic characteristics	
Age, median (IQR)^a^	57 (50–66)
Male (%)	9 (56)
Precedent infection	
Diarrhea (%)	4 (25)
Upper respiratory tract infection (%)	4 (25)
Other (%)	4 (25)
None (%)	4 (25)
Neurological features on admission	
GDS^b^ (%)	
1	4 (25)
2	1 (6.25)
3	2 (12.5)
4	8 (50)
5	1 (6.25)
Sensory deficits (%)	4 (25)
Pain (%)	2 (12.5)
Dysphagia (%)	1 (6.25)
Ventilator dependent (%)	1 (6.25)
Result of CSF analysis (14 patients)	
Median protein level (g/liter, IQR)	0.49 (0.42–0.60)
Increased protein level^c^ (%)	3 (21)
Median white-cell count per mm^3^ (IQR)	2 (2–6)
Treatment	
IVIG (%)	14 (87.5)
Support treatment (%)	2 (12.5)

^a^IQR: interquartile range.

^b^GDS: GBS disability scale scores (Hughes et al.^21^).

^c^The cutoff for elevated CSF protein levels was 0.60 g per liter.

### Cytokine profiles in GBS vs HC

Cytokine profiles were quantified in 5 GBS patients and 5 HC subjects using human 440 cytokine antibodies. Significant differential expression of 67 proteins between GBS patients and HC was demonstrated by the moderated t-statistics test (*p* < 0.05) (Supplementary Table 1).

**Figure 1:**
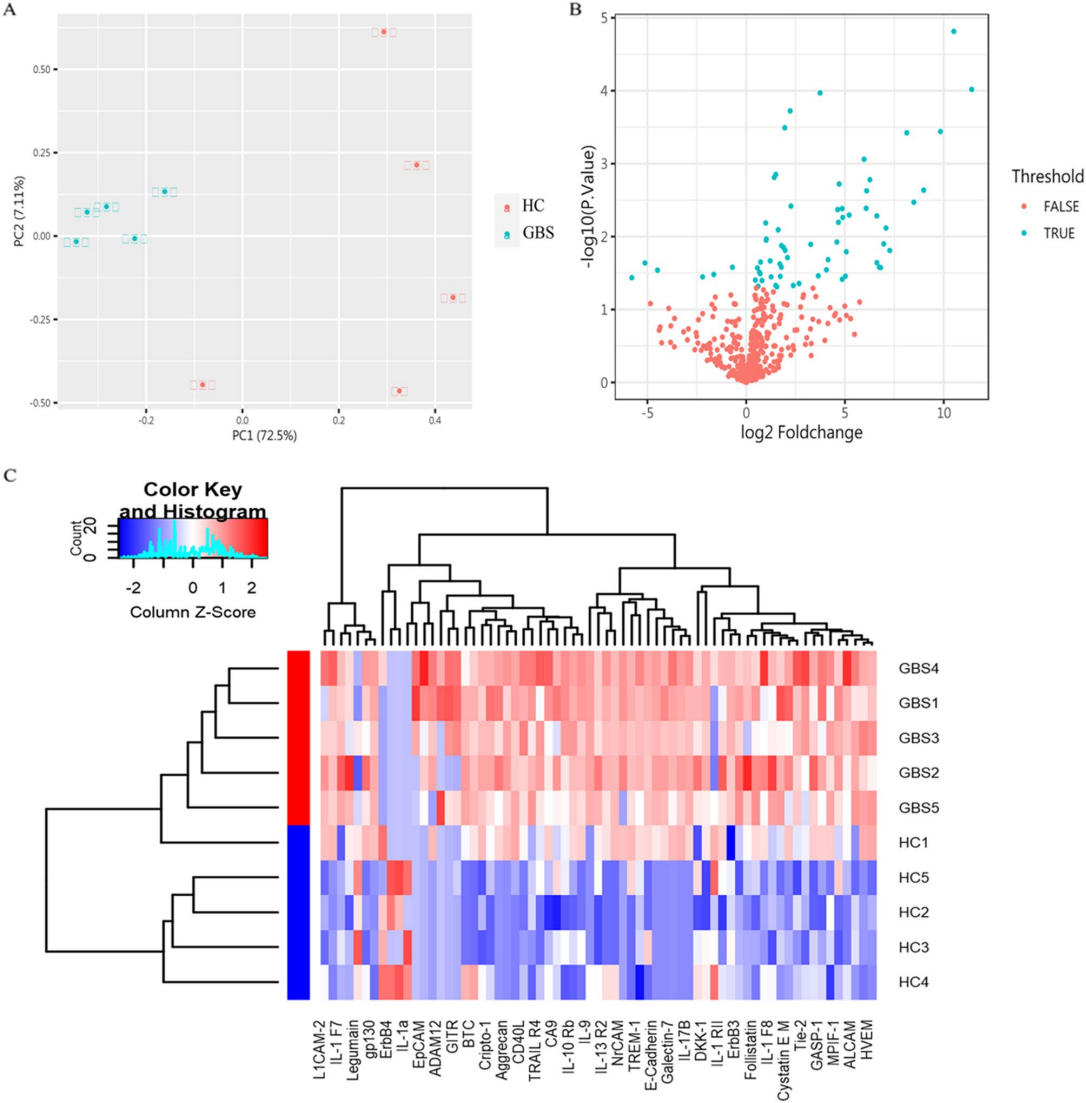
DEPs specificity analysis of GBS. (**A**) Principal component analysis of GBS and HC. A principal component map was drawn to show the differences between the GBS and HC groups (blue for GBS, red for HC). (**B**) Volcano plot of GBS and HC group DEPs screened by Human Antibody Array QAH-CAA-440. DEPs in the GBS and HC groups were obtained based on their log2 FC (x-axis, FC: fold change) and significance (y-axis: − log10 *p*.value). Data set with DEPs with *p* < 0.05 and fold change > 1.2 or < 0.83 are highlighted in blue. (**C**) Heat map of unsupervised hierarchical clustering analysis of DEPs in GBS and HC groups. Based on the average of each protein, low levels are shown in blue, medium levels in white and high levels in red, representing the level of each protein (red for GBS, blue for HC). The heatmap was created using R4.2.3 software (https://cloud.r-project.org/).

First, we used principal component analysis (PCA) for visualization. PCA data revealed that GBS group had a different protein composition compared to the HC group. The first two principal components of 67 DEPs were selected, and the 10 samples were significantly grouped into two clusters by disease status (Fig. 1A). The volcano plot in Fig. 1B shows the distribution of 67 DEPs with a P value less than 0.05. Among the DEPs, there were 61 up-regulation proteins and 6 down-regulation proteins. Finally, we use unsupervised hierarchical cluster analysis to determine whether these DEPs can distinguish between the GBS and HC groups. As shown in Fig. 1C, the results showed an accuracy of 80% in distinguishing the GBS group from the HC group, which further supported the remarkable difference of these proteins between the two groups.

### Expression characteristics of differential proteins

**Figure 2:**
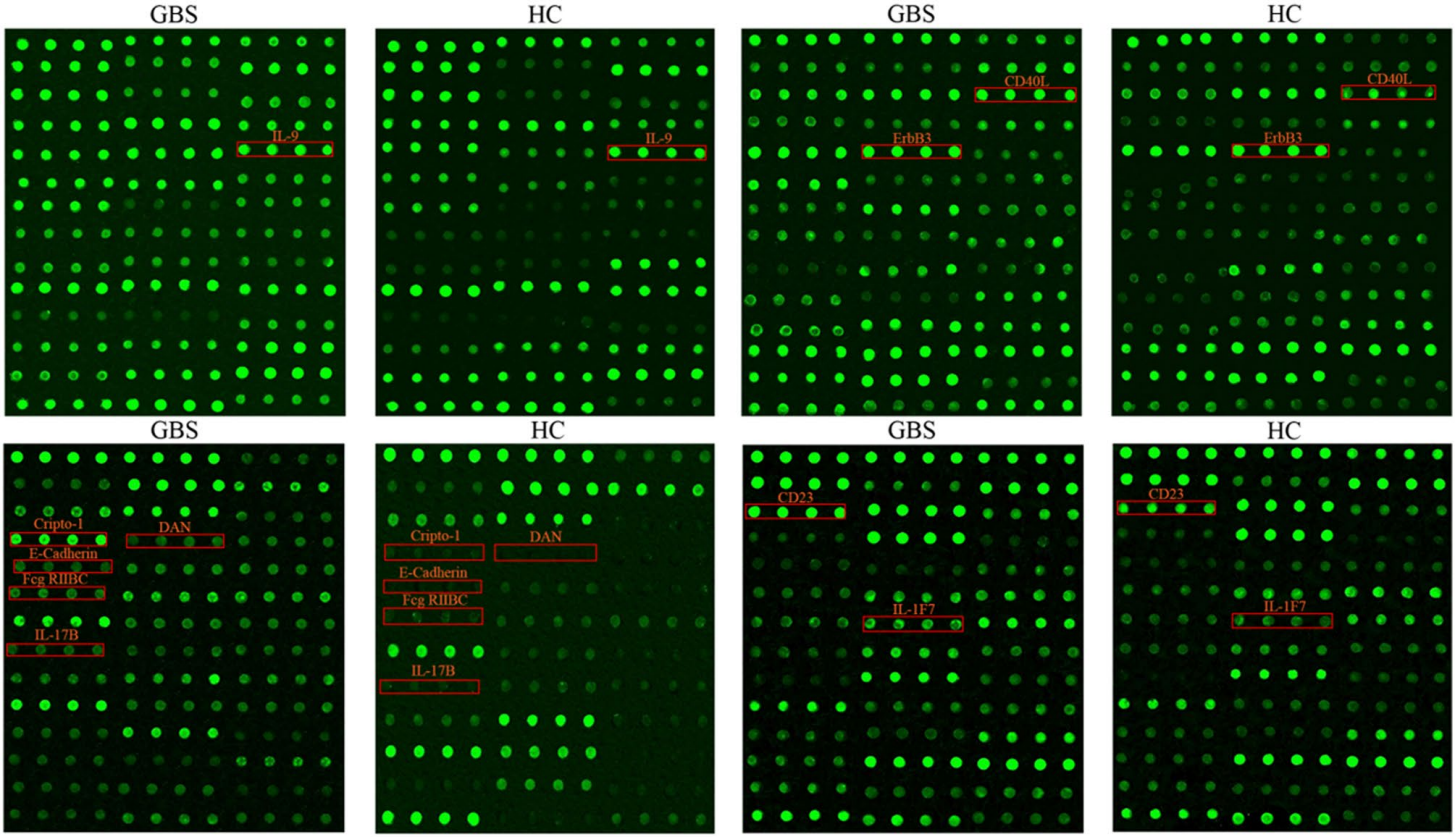
The top 10 DEPs profiles in GBS patients. The top 10 DEPs are marked with red boxes in the QAH-CAA-440 antibody array, each cytokine was repeated four times, and the relationship between fluorescence intensity and cytokine level was shown to be directly proportional.

**Figure 3:**
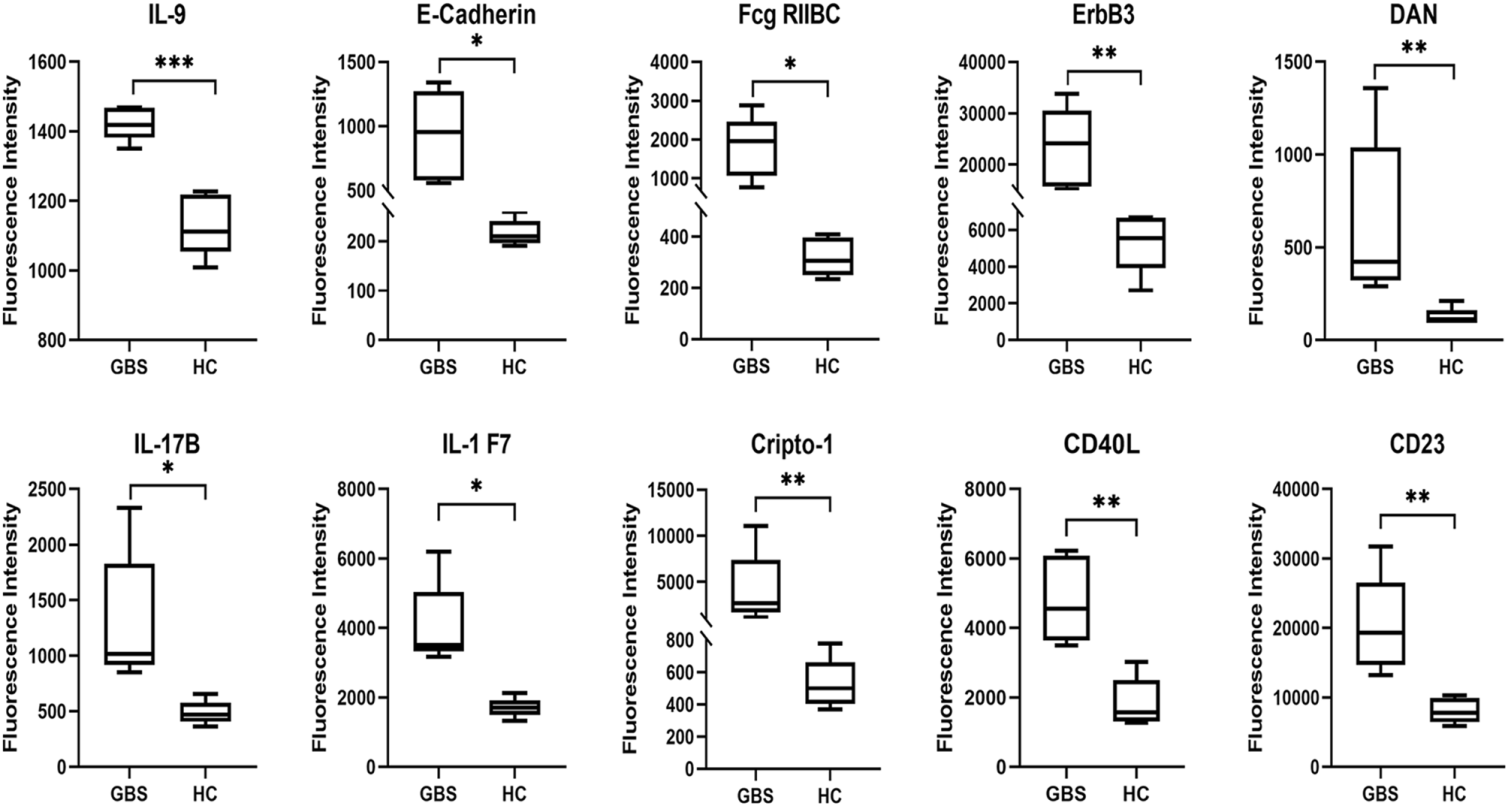
Boxplots were used to analyze the fluorescence intensity of the top 10 DEPs. Every group's midline indicates its average value. *p < 0.05, **p < 0.01.

Among the 67 DEPs, we show a dot plot of antibody arrays for the top 10 differential proteins, where levels of protein are proportional to its fluorescence intensity, as shown in Fig. 2. In addition, the signal values were used to analyze the levels of these proteins by boxplots (Fig. 3).

**Figure 4:**
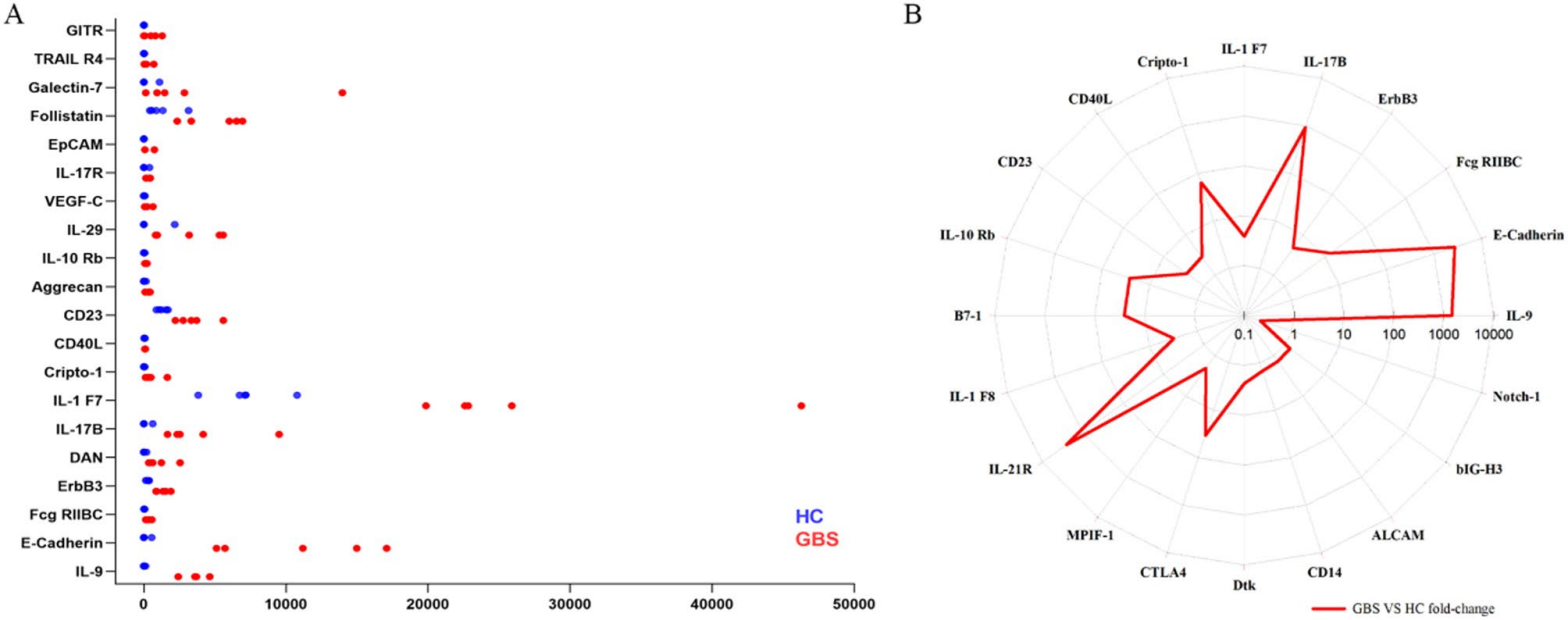
Characteristics of DEPs in GBS. (**A**) Based on the QAH-CAA-440 antibody array screening, the concentration levels of the top 20 DEPs were plotted using horizontal dot plots. The DEPs levels in HC are indicated by blue dots, and those in the GBS patients group are denoted by red dots. (**B**) Based on random forest analysis, the radar plot depicts the fold change of the top 20 serum proteins.

The classification of serum biomarkers that distinguish between the GBS and HC samples was carried out using Random Forests Analysis (RFA), a supervised machine learning technique (Fig. 4B). CD23 was identified using the RFA method, as opposed to Fig. 4A, which was identified using fold change and significance.

### Bioinformatics results

**Figure 5:**
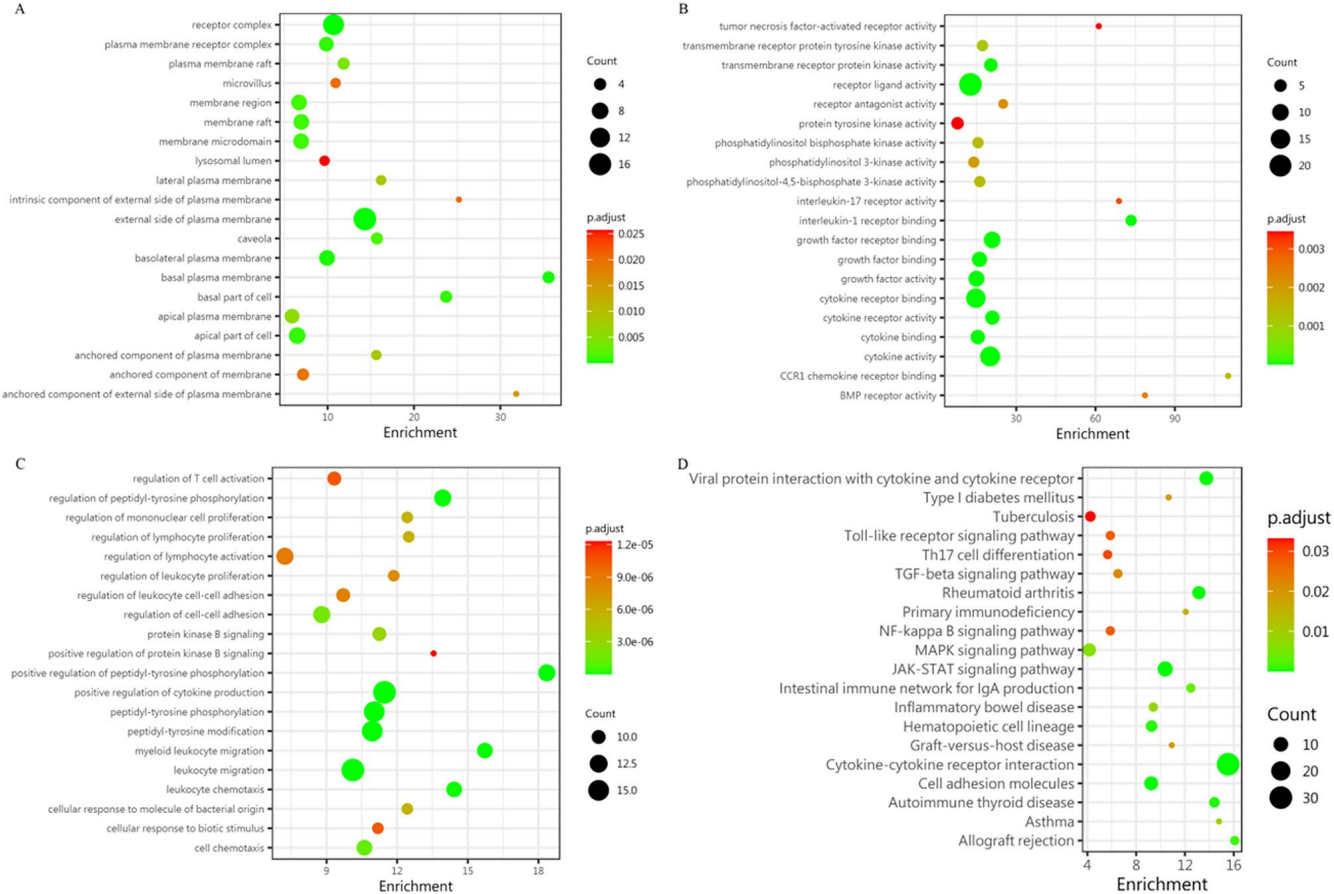
Bioinformatic analysis of differential proteins. (**A**–**C**) Enrichment analysis based on the top 20 cellular components, molecular functions, and biological processes of the GO term. (**D**) Enrichment analysis of the top 20 KEGG pathways. Fisher exact test was used to determine the significance of statistical differences, and *p* < 0.05 was considered to be statistically significant.

**Figure 6:**
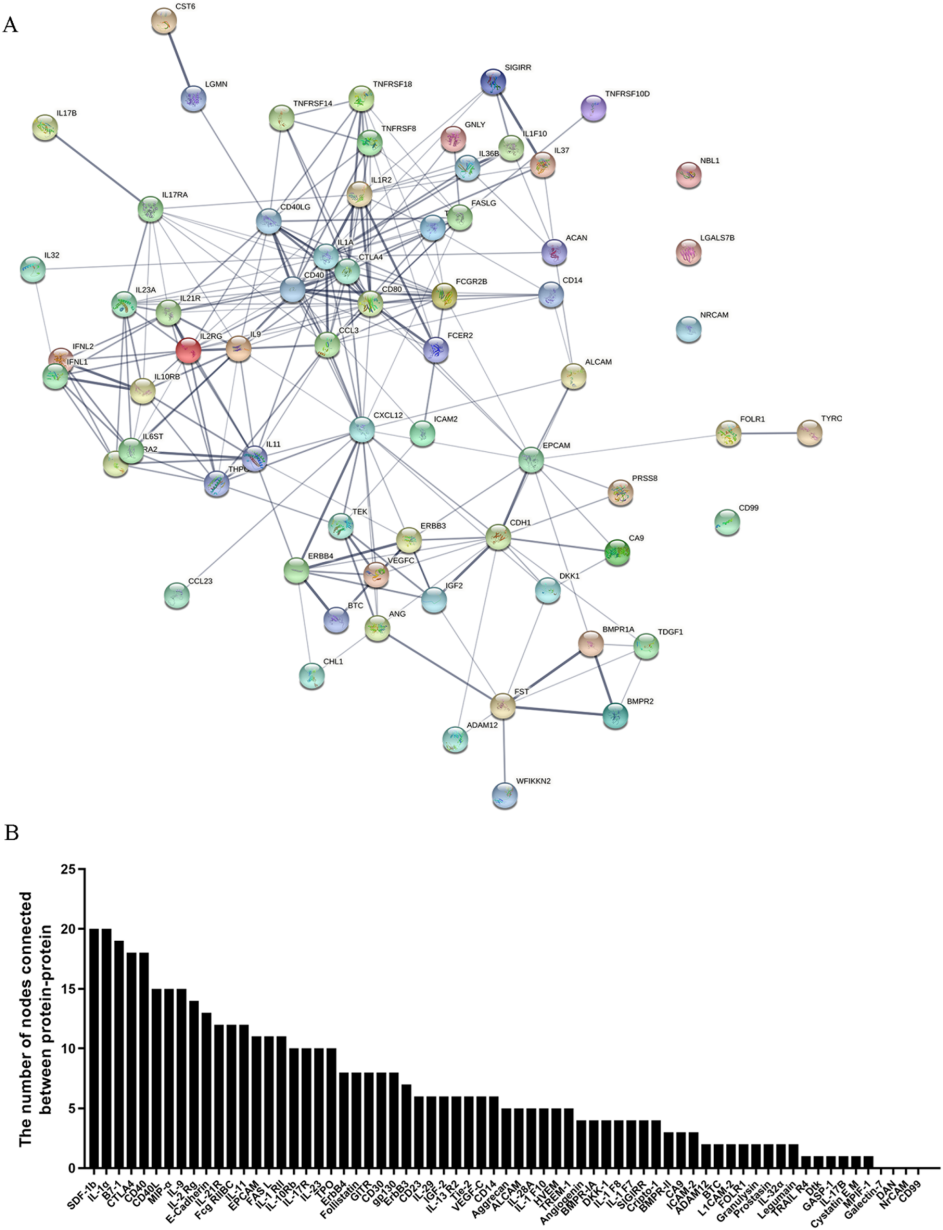
PPI network analysis of DEPs. (**A**) All DEPs were analyzed by the PPI network. The protein–protein line represents the biological function of the correlation, the thickness of the line is strength for protein interactions. (**B**) The number of nodes connected to the DEPs.

To further elucidate the pathogenesis of GBS, GO and KEGG enrichment analysis were performed for DEPs. Figure 5A–C shows the GO enrichment analysis including cellular component, molecular function and biological process, while Fig. 5D shows the KEGG enrichment analysis. Moreover, to further define the more relevant DEPs involved in the pathogenesis of GBS, we created a PPI network to display the interactions of these proteins. Since IL-1α, SDF-1b, B7-1, CD40, CTLA4, IL-9, MIP-1α and CD40L interact with many other proteins, these proteins are considered as central proteins, as shown in Fig. 6. This result suggests that central proteins may be involved in the pathogenesis of GBS by regulating more interacting proteins.

### The result of the antibody array was verified by ELISA

**Figure 7:**
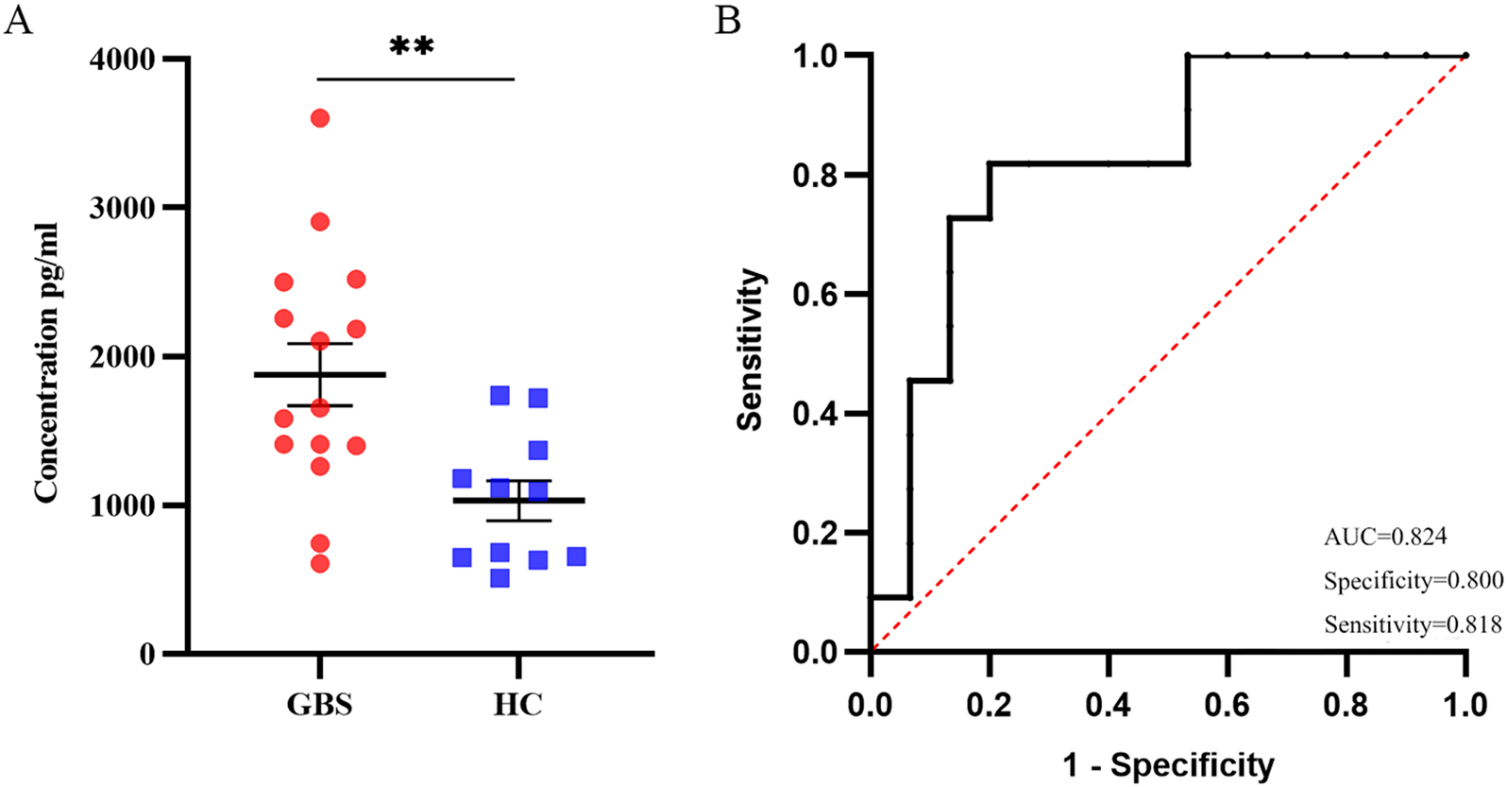
ELISA validation results and statistical analysis. (**A**) Scatter plot of CD23 concentration in GBS and HC groups. ** *p* < 0.01 when compared to HC group. (**B**) The ROC curve analysis of CD23 protein in GBS and HC groups.

To further verify the accuracy of screening DEPs, combined with RFA results, a differential protein was selected for ELISA verification of samples from 26 subjects. The CD23 protein concentration in the GBS group was significantly higher than that in the HC group, as shown in Fig. 7A. The ELISA result was consistent with the array result.

### Sensitivity and specificity analysis of serum protein CD23

The sensitivity, specificity, and area under the ROC curve (AUC) of CD23 protein level in GBS patients were determined by the ROC curve. The AUC for this test is 0.824, the sensitivity is 0.818, and the specificity is 0.800, as shown in Fig. 7B.

## Discussion

It has been reported that the pathogenesis of GBS is related to the interaction between cytokines^22^. In previous studies, the researchers used the Multiplex Suspension Array platform or ELISA to measure the expression levels of chemokines and cytokines in serum, plasma or CSF of patients with GBS^13,23–26^. However, these studies measured only a few dozen chemokines or cytokines, or even fewer, and these studies were unable to find more cytokines involved in the occurrence and development of GBS. Thus, based on antibody array technology consisting of cytokines, chemokines and growth factors, serum levels of 440 proteins were measured in patients with GBS in this study, further explored whether there are more cytokines involved in the pathogenesis of GBS. As a result, there were 67 DEPs were detected between GBS patients and healthy controls, of which 6 proteins were down-regulated and 61 proteins were up-regulated. Among these DEPs, IL-17^27^ and IL-23^28^ were consistent with the previous studies.

Furthermore, more reliable biological information involved in the pathogenesis of GBS can be obtained by understanding more about the functions of DEPs. In consequence, the most abundant GO terms in this study refer to leukocyte, including leukocyte migration, proliferation, chemotaxis, activation, and differentiation, suggesting that the increase of inflammatory lymphocytes and monocytes in circulating blood may play an active role in the inflammatory recruitment of peripheral nerves. Studies have demonstrated that a family of pro-inflammatory molecules, such as chemokines, leukocyte integrins, and cell adhesion molecules are involved in the cascade of leukocyte migration to sites of inflammation^29^. Molecular functional enrichment analysis showed that DEPs mainly focused on the protein activities of receptor ligands, cytokines and growth factors, suggesting that the involvement of these DEPs in the pathogenesis of GBS may be mediated by regulating their protein activities. The biological process function analysis of the GO term revealed that most of the DEPs related to leukocytes were pro-inflammatory cytokines and chemokines, as shown in Table 2. These results suggest that leukocyte proliferation and migration in circulating blood are activated, which may increase the inflammatory recruitment of peripheral nerves, and then lead to the occurrence and development of GBS. KEGG enrichment analysis showed that cytokine-cytokine receptor interaction, JAK-STAT signaling pathway, viral protein interaction with cytokine and cytokine receptor, rheumatoid arthritis, and autoimmune thyroid disease were the most abundant pathways, and studies have confirmed that most pathways are involved in the pathogenesis of GBS^30–33^. Moreover, these most abundant pathways were also confirmed in the analysis of GBS candidate genes^34,35^. Such a result suggested that these pathways may be critical for the occurrence and development of GBS. Further analysis of PPI showed that IL-1α, SDF-1b, B7-1, CD40, CTLA4, IL-9, MIP-1α and CD40L may be the central proteins that mediate the pathological process of GBS by regulating the interaction with other proteins.

**Table 2:** Enrichment of the top 20 biological processes in GO terms.

ID	Description	*p*. adjust	Gene ID
GO:0001819	Positive regulation of cytokine production	1.19E−10	IL9/IL17B/IL37/CD40LG/IFNL1/IL17RA/ TNFRSF8/CD80/IL6ST/IL36B/IL1F10/CD14/TNFRSF14/IL1A/CCL3/CD40/IL23A
GO:0050900	Leukocyte migration	4.46E−10	NBL1/IL37/VEGFC/IL17RA/EPCAM/ TNFRSF10D/TNFRSF18/IL36B/CCL23/TEK/CXCL12/TREM1/IL1F10/LGMN/TNFRSF14/CCL3/IL23A
GO:0050731	Positive regulation of peptidyl-tyrosine phosphorylation	1.22E−09	ERBB3/TDGF1/IFNL1/TNFRSF18/CD80/ IL6ST/IL11/ERBB4/TNFRSF14/CD40/IGF2/ IL23A
GO:0018108	Peptidyl-tyrosine phosphorylation	1.71E−09	ERBB3/TDGF1/IFNL1/TNFRSF18/CD80/ IL6ST/IL11/TEK/TYRO3/BTC/ERBB4/ TNFRSF14/CD40/IGF2/IL23A
GO:0018212	Peptidyl-tyrosine modification	1.71E−09	ERBB3/TDGF1/IFNL1/TNFRSF18/CD80/ IL6ST/IL11/TEK/TYRO3/BTC/ERBB4/ TNFRSF14/CD40/IGF2/IL23A
GO:0050730	Regulation of peptidyl-tyrosine phosphorylation	1.56E−08	ERBB3/TDGF1/IFNL1/TNFRSF18/CD80/ IL6ST/IL11/ERBB4/TNFRSF14/CD40/IGF2/ IL23A
GO:0097529	Myeloid leukocyte migration	2.53E−08	NBL1/IL37/VEGFC/IL17RA/IL36B/CCL23/ CXCL12/IL1F10/LGMN/CCL3/IL23A
GO:0030595	Leukocyte chemotaxis	5.59E−08	NBL1/IL37/VEGFC/IL17RA/IL36B/CCL23/ CXCL12/IL1F10/LGMN/CCL3/IL23A
GO:0060326	Cell chemotaxis	1.26E−06	NBL1/IL37/VEGFC/IL17RA/IL36B/CCL23/ CXCL12/IL1F10/LGMN/CCL3/IL23A
GO:0022407	Regulation of cell–cell adhesion	1.76E−06	CDH1/FCGR2B/CD40LG/IFNL1/EPCAM/ CD80/IL6ST/CXCL12/CTLA4/TNFRSF14/ IGF2/IL23A
GO:0043491	Protein kinase B signaling	3.12E−06	ERBB3/CD80/TEK/TYRO3/THPO/BTC/ ERBB4/CCL3/CD40/IGF2
GO:0050670	Regulation of lymphocyte proliferation	5.70E−06	FCGR2B/CD40LG/CD80/IL6ST/CTLA4/ TNFRSF14/CD40/IGF2/IL23A
GO:0032944	Regulation of mononuclear cell proliferation	5.70E−06	FCGR2B/CD40LG/CD80/IL6ST/CTLA4/ TNFRSF14/CD40/IGF2/IL23A
GO:0071219	Cellular response to molecule of bacterial origin	5.70E−06	FCGR2B/IL37/CD80/IL36B/IL1F10/CD14/ CCL3/CD40/SIGIRR
GO:0070663	Regulation of leukocyte proliferation	8.00E−06	FCGR2B/CD40LG/CD80/IL6ST/CTLA4/ TNFRSF14/CD40/IGF2/IL23A
GO:1903037	Regulation of leukocyte cell–cell adhesion	8.57E−06	FCGR2B/CD40LG/IFNL1/CD80/IL6ST/ CXCL12/CTLA4/TNFRSF14/IGF2/IL23A
GO:0051249	Regulation of lymphocyte activation	8.92E−06	FCGR2B/CD40LG/IFNL1/TNFRSF18/CD80/ IL6ST/CTLA4/TYRO3/TNFRSF14/CD40/ IGF2/IL23A
GO:0071216	Cellular response to biotic stimulus	1.05E−05	FCGR2B/IL37/CD80/IL36B/IL1F10/CD14/ CCL3/CD40/SIGIRR
GO:0050863	Regulation of T cell activation	1.05E−05	FCGR2B/CD40LG/IFNL1/TNFRSF18/CD80/ IL6ST/CTLA4/TNFRSF14/IGF2/IL23A
GO:0051897	Positive regulation of protein kinase B signaling	1.23E−05	ERBB3/CD80/TEK/THPO/BTC/ERBB4/ CCL3/IGF2

IL-1α is a ubiquitous and pivotal pro-inflammatory cytokine that, like IL-1β, belongs to the IL-1 family. Studies have reported an increase in the blood level of the specific cytokine IL-1β when comparing GBS patients with healthy controls^36^. However, Debnath, M., et al. reported that plasma IL-1β levels were significantly lower in GBS patients than in healthy controls^13^. Sivieri., et al. confirmed that IL-1α was not found in the CSF from GBS patients^37^, but there have been no reports on the abnormal level of IL-1α in the serum of GBS patients and the specific pathogenesis of IL-1α in GBS. MIP-1α (CCL3) and SDF-1b (CXCL12b) are chemokines in these central proteins. Studies have confirmed that chemokines play a prominent role in the recruitment of leukocytes to inflamed tissues^29^. MIP-1α/CCL3 is involved in the recruitment of monocytes to EAN rats PNS, and MIP-1α neutralizing antibody inhibits the occurrence of neurological signs by significantly inhibiting sciatic nerve macrophages and T cells^38^. And more importantly, one study showed peak up-regulation of MIP-1α mRNA at day 13 (p.i.) after immunization, just before the most severe disease^39^. Our study found that serum levels of IL-1α and MIP-1α were decreased in patients with GBS. Their down-regulation indicates that these two proteins may play an anti-inflammatory role in the primary immune pathogenesis of GBS, possibly through ligand and receptor interactions, particularly in the cytokine-cytokine receptor interaction, which is consistent with pathway analysis. However, more studies are needed to verify and elucidate these effects. An increase in chemokines, such as CXCL12, in the CSF of GBS patients has been reported^40^. Moreover, in EAN, CXCL12 is particularly relevant for the regulation of leukocyte homing to the sciatic nerve by binding to its receptor CCR7^41^. In this study, the expression of CXCL12b was elevated in GBS serum, which suggested that CXCL12b might be a potential serum biomarker of GBS. B7-1 (CD80) provides a costimulatory signal for T-cell activation^42^, and overexpression of the costimulatory molecule CD80 can mediate autoimmune diseases^43^. Previous studies have identified a second receptor for B7 on T cells as cytotoxic T lymphocyte-associated molecule-4 (CTLA-4). CTLA4 is expressed on activated T cells and subsequently induces a negative down-regulation response to maintain immune homeostasis. Mice lacking CD80 or anti-CTLA-4 antibody treatment had very low neurological signs and resistance to EAN-induced resistance, and markedly reduced inflammatory infiltrates in target the tissues^44,45^. In this study, the expression of B7-1 and CTLA-4 in serum of GBS patients was up-regulated, suggesting that they may be potential serum biomarkers and important therapeutic targets for GBS. CD40 and CD40L are a pair of costimulatory molecules, the expression of CD40 on plasmacytoid dendritic cells surface in the acute phase of GBS was up-regulated^46^. An article reported that B cells exert a synergistic effect through CD40L-CD40 interaction in T cell-mediated EAN of Lewis rats^47^. In mice EAN, therapeutic administration of anti-CD40 prevented the full activation of CD4-positive T cells during priming and inhibited the production of IFN-γ in peripheral lymph nodes, spleen, and serum, as well as the production of IL-6, IL-12p40, intercellular adhesion molecule-1 (ICAM-1), and vascular cell adhesion molecule-1 (VCAM-1) associated with the activation of NF-κB signaling pathway, which underscores the functional importance of CD40-mediated EAN immune responses in mice^48^. A study has confirmed that CD40L is a potential plasma biomarker during the acute phase of GBS, which is related to the pathogenesis of GBS^49^. PPI results of this study, CD40 interacts with 18 proteins, including IL-23A, IL-1α, TNFRSF8, FCER2, CD80, IL-9, CTLA4, CD14, IL-17RA, TNFRSF18, IL-21R, FCGR2B, FAS-L, CD40L, IL-2RG, ICAM2, CXCL12 and CCL3. GO analysis showed that CD40, CD40L, CTLA4, IL-23A, CD80 and FCGR2B were co-involved in lymphocyte, monocyte and leukocyte proliferation and lymphocyte activation. Further analysis of KEGG pathway enrichment suggested that CD40, CD40L, CXCL12 and CD14 were involved in NF-κB signaling pathway. In conclusion, bioinformatics analysis suggested that CD40 may activate NF-κB signaling pathway by mediating related proteins in leukocytes, monocytes and lymphocytes, and then induce the occurrence and development of GBS. Therefore, CD40 may be a potential biomarker for GBS and a suitable potential target for the treatment of GBS. IL-9 is a pro-inflammatory factor associated with autoimmune diseases. IL-9 is expressed in the CSF of patients with multiple sclerosis (MS) and inversely correlated with disease severity^50^. Previous studies have demonstrated that IL-9 is expressed in the brain of patients with progressive MS, which is involved in the immune mechanism of progressive MS by regulating microglia and macrophages^51^. However, no studies on IL-9 in GBS have been reported. In the present study, the IL-9 protein level in the GBS group was significantly higher than that in the healthy control group, suggesting that IL-9 may also be a serum biomarker of GBS. CD23 is the FC-epsilon receptor, which is expressed in T, B lymphocytes and monocytes, and its expression is regulated by a variety of stimuli^52^. Some research has suggested that CD23 is expressed by M2 anti-inflammatory macrophages and therefore may be involved in the anti-inflammatory response^53,54^. One study showed an increased ratio of CD23-positive monocytes/macrophages in peripheral blood from patients with acute relapsing MS^55^, which may serve as a marker of tissue repair^56^. Thus far, there is no report on the effect of CD23 on GBS. This research found that the expression of CD23 protein in GBS serum was increased compared with healthy people, indicating that it may be a novel serum biomarker for GBS. However, further research is needed to confirm and elucidate its specific role and pathogenesis in GBS. Taken together, these proteins may be relatively reliable serum biomarkers of GBS, and intervention treatment of them may reduce the occurrence and delay the development of GBS.

In summary, we found that 61 cytokines were higher and 6 cytokines were lower in the serum of GBS patients than in healthy controls. Based on the results of bioinformatics analysis, we speculated that the activation of leukocyte proliferation and migration in circulating blood may play a proactive role in the inflammatory recruitment of peripheral nerves, thereby inducing GBS, but further verification is needed. In addition, we also found that the central proteins in GBS serum are IL-1α, SDF-1b, B7-1, CD40, CTLA4, IL-9, MIP-1α and CD40L, which may play a dominant role in the immune pathogenesis of GBS through more interactions with other proteins. And, more importantly, IL-1α, IL-9 and CD23 were first detected in the serum of patients with GBS, suggesting they may be promising serum biomarkers for the treatment of GBS. Besides, this study is exploratory and has certain limitations due to its small sample size. Thus, a larger sample size is needed for clinical validation, and cell and animal experiments to study its pathogenesis.

## Materials and methods

### Subjects

**Figure 8:**
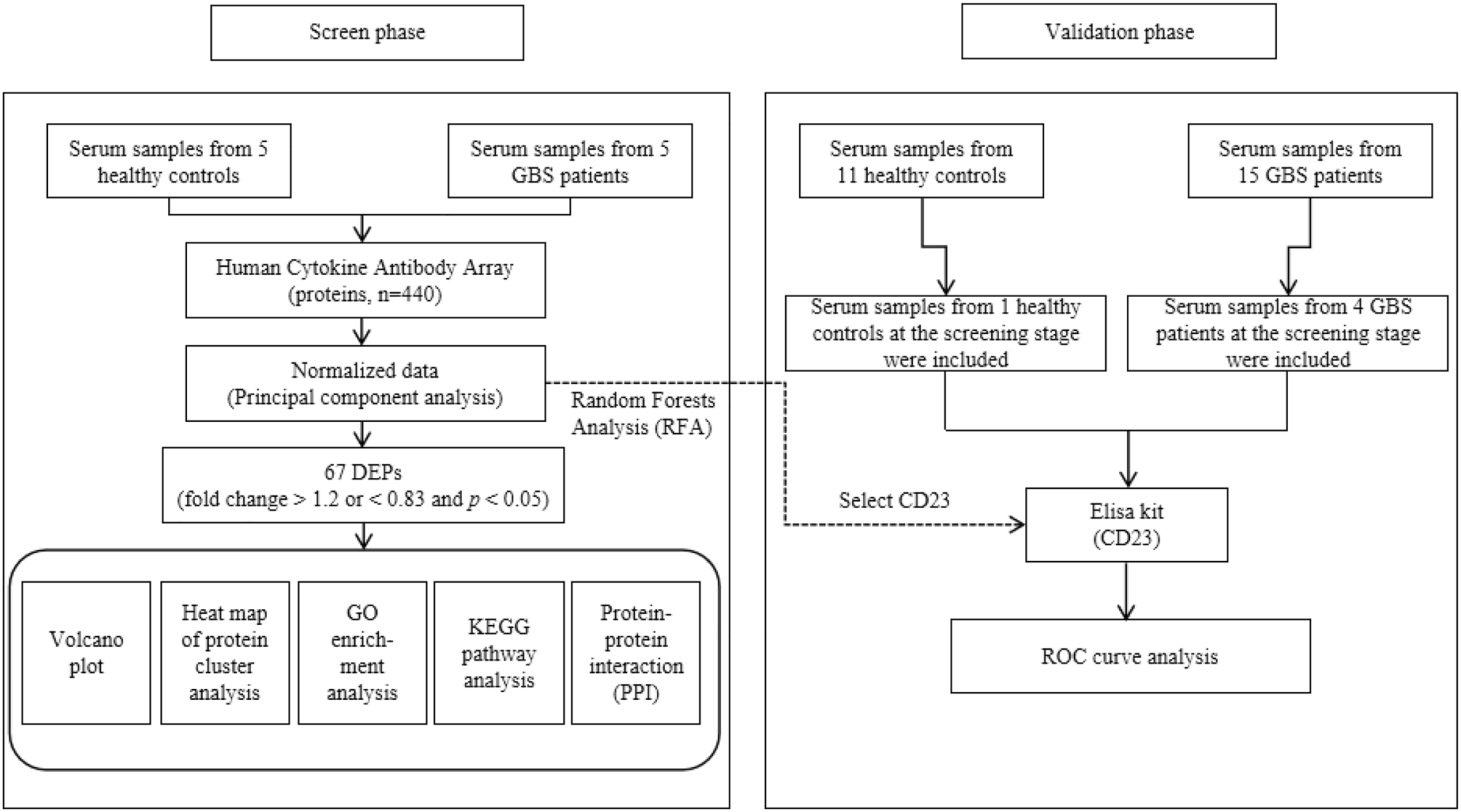
Flowchart of this study.

31 subjects were recruited in two stages at the General Hospital of Ningxia Medical University. In the screening phase, 10 subjects, namely: 5 confirmed GBS patients and 5 age-and sex-matched HCs were enrolled. In the validation phase, 26 subjects, namely: 15 GBS patients and 11 age-and sex-matched hc, were enrolled. In this cohort, 5 subjects from the screening phase were included (Fig. 8). All patients were in accordance with the 1990 Asbury and Cornblath diagnostic criteria^57^. Patients with the following conditions were excluded: (1) acute onset of CIDP is likely to be diagnosed, that is, if the patient has another exacerbation 8 weeks after the onset, or if the exacerbation has occurred 3 or more times; (2) patients with autoimmune diseases such as MS, IBD, RA, type 1 diabetes, or malignant tumors; (3) recent infection or failure to collect serum samples during the acute phase (within 3–5 days from onset of symptoms to admission) and prior to intravenous immunoglobulin (IVIG) treatment. The control group was healthy people without recent or chronic infections who underwent physical examination in the Department of Neurology, General Hospital of Ningxia Medical University. All 31 subjects were negative for COVID-19. All qualified subjects' blood samples were collected. Blood samples were centrifuged at 2000 r/min at 4 ℃ for 10 min, serum was collected and stored at − 80 ℃ until further experiments.

Blood samples were collected with the consent of the patients and signed informed consent. This study was in accordance with the Declaration of Helsinki and was approved by the Medical Ethics Committee of Ningxia Medical University General Hospital (protocol number 2018-318).

### Quantibody array performance

440 cytokines were measured by a sandwich method based on the antibody array technique (Human Cytokine Antibody Array, RayBiotech, Norcross, GA, USA). Briefly, 100ul of serum was added to a chip containing 440 primary antibodies after 1:2 dilution of the serum sample and incubated at 4℃ overnight incubation. The chips were first washed 10 times with wash I diluted with deionized water for 10 s each shake, and then cleaned 6 times with wash II diluted with deionized water for 10 s each shake, then, biotinylated secondary antibodies were added and incubated for 2 h on a shaker at room temperature. After cleaning the chip again using the method described above, Cy3-conjugated streptavidin was added and incubated at room temperature in the dark for 1 h. InnoScan 300 Microarray scanner (Innopsys) is used to measure the fluorescent signal values. Then, using Mapix software will fluorescent the signal value into quantitative values.

### ELISA performance

In order to further verify the screening results, according to the manufacturer's instructions (RayBiotech, Norcross GA, USA), a CD23 ELISA test was performed. In addition, we increased the number of subjects to 26 to improve the accuracy of validation. Briefly, serum was added to 96-well plates after dilution according to instructions and incubated overnight at 4 °C. The 96-well plates were washed five times using a wash solution diluted with deionized water, each with shaking for 1 min at room temperature, then added biotin-conjugated antibody, and incubated for 2 h. HRP-conjugated streptavidin was added after washing the plates again using the method described above, followed by the addition of TMB One-Step Substrate Reagent. The OD450 value was measured using an ELx800NB plate reader (Biotek).

### Statistical analysis

The data were plotted and analyzed using the limma package from R/Bioconductor or GraphPad Prism 8.0.2 software. Data are presented as mean ± standard deviation. The data of the human cytokine antibody array were analyzed by the moderated t-statistical test, fold change > 1.2 or < 0.83 and *P* < 0.05 was considered statistically significant. For ELISA data, a Student's t-test was used where the samples passed the normality test, otherwise, the Mann Whitney U test was used to analyze the data. The ROC curve was drawn to determine the ability of the serum biomarker to distinguish GBS patients from healthy controls.

### Bioinformatics analysis

Based on the enrichment analysis of GO terms and KEGG^58–60^, the potential functions of the DEPs associated with GBS were further elucidated by describing the cellular components, molecular functions and biological processes of these proteins. A significant association with GBS pathogenesis was defined as an adjusted *P* < 0.05. The STRING database https://string-db.org/) was used for PPI analysis, and the node proteins involved in protein–protein interaction were identified by protein ID.

To ensure the relative importance of each serum protein in the classification of GBS and healthy control group, we used an R-executed dimensionality reduction machine learning algorithm, namely: Random Forest Classification analysis. Based on the method of random forest classification analysis, Excel was used to plot the fold changes of the top 20 serum proteins between GBS and healthy controls as radial plots.

## Supplementary Information

Supplementary Information.

## Data Availability

Data from this study are included in this paper and its Supplementary material, and all digits and decimals are correct and reliable. Additional data can be requested directly from the corresponding author.
