# Supplementary material for: Extensive cytokine biomarker analysis in serum of Guillain-Barré syndrome patients
Source: Sci Rep. 2023 May 23;13:8354. doi: 10.1038/s41598-023-35610-w (PMC10205034; doi:10.1038/s41598-023-35610-w)
Supplement: Supplementary file 1 — Supplementary Information. [file 41598_2023_35610_MOESM1_ESM.docx]

**S1.67 differential proteins were detected in GBS and HC controls**

| **Protein ID** | **Entrez ID** | **Uniprot ID** | **AveExp.GBS** | **AveExp.HC** | **GBS/HC** | | **Regulation** |
| --- | --- | --- | --- | --- | --- | --- | --- |
|  |  |  |  |  | ***P* value Fold change** | |  |
| IL-9 | 3578 | P15248 | 11.85 | 1.34 | ＜0.001 | 1459.68 | up |
| E-Cadherin | 999 | P12830 | 13.24 | 1.82 | ＜0.001 | 2734.35 | up |
| Fcg RIIBC | 2213 | P31994 | 8.21 | 4.47 | ＜0.001 | 13.36 | up |
| ErbB3 | 2065 | P21860 | 10.28 | 8.05 | ＜0.001 | 4.68 | up |
| DAN | 4681 | P41271 | 9.628 | 1.49 | ＜0.001 | 280.07 | up |
| IL-17B | 27190 | Q9UHF5 | 11.69 | 1.86 | ＜0.001 | 911.07 | up |
| IL-1 F7 | 27178 | Q9NZH6 | 14.68 | 12.72 | ＜0.001 | 3.87 | up |
| Cripto-1 | 6997 | P13385 | 8.59 | 2.63 | ＜0.001 | 62.57 | up |
| CD40L | 959 | P29965 | 6.64 | 5.14 | 0.001 | 2.86 | up |
| CD23 | 2208 | P06734 | 11.71 | 10.29 | 0.002 | 2.67 | up |
| Aggrecan | 176 | P16112 | 7.68 | 1.42 | 0.002 | 76.80 | up |
| IL-10 Rb | 3588 | Q08334 | 7.00 | 2.30 | 0.002 | 26.08 | up |
| IL-29 | 282618 | Q8IU54 | 11.20 | 2.22 | 0.002 | 507.02 | up |
| VEGF-C | 7424 | P49767 | 7.28 | 1.19 | 0.002 | 68.37 | up |
| IL-17R | 23765 | Q96F46 | 8.13 | 2.06 | 0.004 | 67.36 | up |
| EpCAM | 4072 | P16422 | 4.86 | 0 | 0.004 | 29.04 | up |
| Follistatin | 10468 | P19883 | 12.17 | 9.91 | 0.004 | 4.81 | up |
| Galectin-7 | 3963 | P47929 | 10.50 | 2.02 | 0.003 | 358.18 | up |
| TRAIL R4 | 8793 | Q9UBN6 | 6.77 | 2.14 | 0.004 | 24.75 | up |
| GITR | 8784 | Q9Y5U5 | 6.89 | 0.27 | 0.005 | 97.80 | up |
| FAS L | 356 | P48023 | 7.49 | 2.28 | 0.005 | 37.11 | up |
| CD30 | 943 | P28908 | 4.89 | 0 | 0.005 | 29.58 | up |
| B7-1 | 941 | P33681 | 7.62 | 2.95 | 0.006 | 25.41 | up |
| gp130 | 3572 | P40189 | 13.53 | 12.54 | 0.007 | 1.98 | up |
| IL-13 R2 | 3598 | Q14627 | 10.56 | 3.49 | 0.008 | 134.88 | up |
| IL-1 F8 | 27177 | Q9NZH7 | 11.70 | 10.08 | 0.008 | 3.07 | up |
| MPIF-1 | 6368 | P55773 | 10.36 | 9.34 | 0.011 | 2.03 | up |
| ICAM-2 | 3384 | P13598 | 17.35 | 16.34 | 0.011 | 2.02 | up |
| IL-32 alpha | 9235 | P24001 | 7.90 | 3.31 | 0.012 | 24.09 | up |
| IL-21R | 50615 | Q9HBE5 | 10.86 | 3.89 | 0.013 | 124.66 | up |
| IL-11 | 3589 | P20809 | 4.91 | 1.64 | 0.013 | 9.62 | up |
| Tie-2 | 7010 | Q02763 | 10.43 | 8.64 | 0.013 | 3.45 | up |
| BMPR-IA | 657 | P36894 | 10.75 | 8.86 | 0.014 | 3.71 | up |
| SDF-1b | 6387 | P48061 | 11.51 | 4.24 | 0.016 | 153.93 | up |
| GASP-1 | 124857 | Q8TEU8 | 11.24 | 9.27 | 0.015 | 3.92 | up |
| CTLA4 | 1493 | P16410 | 9.28 | 4.22 | 0.016 | 33.52 | up |
| Prostasin | 5652 | Q16651 | 11.65 | 9.56 | 0.019 | 4.26 | up |
| TREM-1 | 54210 | Q9NP99 | 9.56 | 5.41 | 0.021 | 17.71 | up |
| Dtk | 7301 | Q06418 | 9.58 | 8.36 | 0.022 | 2.32 | up |
| Angiogenin | 283 | P03950 | 10.52 | 9.79 | 0.022 | 1.66 | up |
| TPO | 7066 | P40225 | 10.76 | 4.15 | 0.023 | 98.11 | up |
| FOLR1 | 2348 | P15328 | 1.08 | 6.20 | 0.023 | 0.03 | down |
| IL-1 F10 | 84639 | Q8WWZ1 | 15.21 | 13.51 | 0.024 | 3.26 | up |
| CD14 | 929 | P08571 | 11.52 | 10.95 | 0.027 | 1.48 | up |
| DKK-1 | 22943 | O94907 | 13.18 | 6.44 | 0.026 | 106.82 | up |
| NrCAM | 4897 | Q92823 | 8.51 | 1.70 | 0.027 | 111.85 | up |
| Legumain | 5641 | Q99538 | 13.14 | 13.83 | 0.026 | 0.62 | down |
| BMPR-II | 659 | Q13873 | 11.23 | 9.46 | 0.026 | 3.43 | up |
| BTC | 685 | P35070 | 6.70 | 2.64 | 0.029 | 16.68 | up |
| ErbB4 | 2066 | Q15303 | 0 | 4.49 | 0.029 | 0.04 | down |
| HVEM | 8764 | Q92956 | 10.67 | 10.00 | 0.031 | 1.60 | up |
| IL-2 Rg | 3561 | P31785 | 1.72 | 0 | 0.035 | 3.31 | up |
| IL-1a | 3552 | P01583 | 0 | 1.64 | 0.033 | 0.32 | down |
| MIP-1a | 6348 | P10147 | 8.27 | 10.48 | 0.036 | 0.22 | down |
| CD40 | 958 | P25942 | 4.62 | 0.98 | 0.034 | 12.52 | up |
| L1CAM-2 | 10752 | O00533 | 16.19 | 14.93 | 0.036 | 2.40 | up |
| ADAM12 | 8038 | O43184 | 5.02 | 0 | 0.035 | 32.51 | up |
| CD99 | 4267 | P14209 | 10.31 | 9.60 | 0.032 | 1.63 | up |
| IGF-2 | 3481 | P01344 | 0 | 5.79 | 0.037 | 0.02 | down |
| SIGIRR | 59307 | Q6IA17 | 9.59 | 4.73 | 0.039 | 29.03 | up |
| ALCAM | 214 | Q13740 | 10.80 | 10.34 | 0.039 | 1.38 | up |
| Granulysin | 10578 | P22749 | 13.04 | 12.24 | 0.040 | 1.74 | up |
| CA9 | 768 | Q16790 | 6.38 | 3.71 | 0.044 | 6.39 | up |
| IL-28A | 282616 | Q8IZJ0 | 5.46 | 3.07 | 0.047 | 5.22 | up |
| IL-1 RII | 7850 | P27930 | 9.24 | 7.74 | 0.047 | 2.82 | up |
| Cystatin E M | 1474 | Q15828 | 11.24 | 10.64 | 0.048 | 1.52 | up |
| IL-23 | 51561 | Q9NPF7 | 12.27 | 10.73 | 0.049 | 2.93 | up |
